# Dietary Beta-Hydroxy-Beta-Methyl Butyrate Supplementation Inhibits Hepatic Fat Deposition via Regulating Gut Microbiota in Broiler Chickens

**DOI:** 10.3390/microorganisms10010169

**Published:** 2022-01-13

**Authors:** Shiyu Zhang, Zhiyi Tang, Changbing Zheng, Yinzhao Zhong, Jie Zheng, Geyan Duan, Yulong Yin, Yehui Duan, Zehe Song

**Affiliations:** 1 CAS Key Laboratory of Agro-Ecological Processes in Subtropical Region, Hunan Provincial Key Laboratory of Animal Nutritional Physiology and Metabolic Process, National Engineering Laboratory for Pollution Control and Waste Utilization in Livestock and Poultry Production, Institute of Subtropical Agriculture, Chinese Academy of Sciences, Changsha 410125, China; zhangshiyu211@mails.ucas.ac.cn (S.Z.); chamdpion@163.com (C.Z.); yinzhaoz@163.com (Y.Z.); zhengjie202@mails.ucas.ac.cn (J.Z.); duangeyan21@mails.ucas.ac.cn (G.D.); yinyulong@isa.ac.cn (Y.Y.); 2 College of Advanced Agricultural Sciences, University of Chinese Academy of Sciences, Beijing 100039, China; 3 Animal Nutritional Genome and Germplasm Innovation Research Center, College of Animal Science and Technology, Hunan Agricultural University, Changsha 410128, China; zytangd@163.com

**Keywords:** broilers, beta-hydroxy-beta-methyl butyrate, fat deposition, gut microbiota

## Abstract

The present study is aimed to explore the effects of different dietary beta-hydroxy-beta-methyl butyrate (HMB) levels (0, 0.05%, 0.10%, or 0.15%) on liver lipid metabolism on Wenshi broiler chickens. Results showed that HMB reduced the liver weight as well as liver concentrations of triacylglycerol (TG) and total cholesterol (TC) (quadratically, *p* < 0.05), and the lowest values were observed in the 0.10% HMB group. Meanwhile, HMB supplementation significantly altered the expression levels of key genes related to lipid metabolism in the liver of broiler chickens (*p* < 0.05). Furthermore, 16S rRNA gene sequencing revealed that HMB supplementation could greatly change the richness, diversity, and composition of the broiler gut microbiota, and the *Bacteroidetes* relative abundance at the phylum level and the *Alistipes* relative abundance at the genus level were affected (*p* < 0.05). Correlation analysis further suggested a strong association between *Bacteroidetes* relative abundance and lipid metabolism-related parameters (*p* < 0.05). Together, these data suggest that 0.10% HMB supplementation could inhibit hepatic fat deposition via regulating gut microbiota in broilers.

## 1. Introduction

In recent years, with the improvement of living standards, public demands for safe, healthy, and high-quality meat in human diets are increasing [1]. Broiler chickens, as the most commonly farmed animals in the world, not only provide animal protein for human growth and development, but also serve as powerful experimental models for basic and applied research [2]. However, the rapid growth of modern broilers can increase the liver load, thus leading to a higher risk of fat accumulation in the liver [3]. For chickens, unlike mammals, the liver is the primary site for de novo fatty acid synthesis and synthetic fat is mainly deposited in the adipose tissue [4,5]. The increase of liver fat content will undoubtedly lead to the increase of lipid peroxidation and steatosis, and may even lead to fatty liver hemorrhage syndrome. The increase of fat deposition in peripheral tissues (especially the abdominal adipose tissue) will lead to a reduction in meat quality and an increase in feed cost [6]. Surprisingly, excessive abdominal fat deposition-induced economic losses are estimated to be over $2.7 billion [2]. In this context, one effective way to reduce economic losses of many poultry producers is to inhibit fat synthesis and deposition in broiler chickens. Given that the genetic selection against fatness is slow, there has been a growing focus on the nutritional regulation of fatty acid synthesis and fat deposition in broiler chickens [7,8].

Fat metabolism, including the processes of digestion, absorption, synthesis, and catabolism, is an essential and complex biochemical reaction [9]. There is evidence showing that fat deposition can be altered by modulating the gene expression implicated in liver fatty acid metabolism, such as fatty acid synthesis-related genes (acetyl-CoA carboxylase, *ACC*; sterol regulatory element-binding protein-1c, *SREBP-1c*) and lipolysis-related genes (acyl-CoA oxidase 1, *ACOX1*; carnitine palmitoyl transferase I, *CPT-I*, lipoprotein lipase, *LPL*; peroxisome proliferator-activated receptor α, *PPARα*) [7,10]. In addition, slaughter performance in poultry can be improved by effectively regulating the expression of lipid metabolism-related genes in the liver [11,12]. Previous studies have demonstrated that β-hydroxy-β-methylbutyrate (HMB), a metabolite of leucine, could augment body weight gain and slaughter performance in broiler chickens [13,14]. Moreover, recent evidence from rodent and swine models suggests that HMB administration could reduce fat deposition [15,16]. Therefore, we hypothesized that HMB might effectively mitigate fat deposition in broiler chickens, thus reducing the economic losses of many poultry producers.

Evidence from rodent models has suggested that the beneficial effects of HMB in fat deposition are likely mediated by improvements to gut microbiota composition [15]. Due to the unique digestive system and gut microbiota composition of chickens, the regulatory mechanism of action of HMB observed in rodent models might not be suitable for poultry. Further investigation is necessary to elucidate the role and mechanisms of HMB in the fat metabolism of broiler chickens. Therefore, this study aimed to explore the mechanism of HMB on fat deposition, the hepatic expression of fat metabolism-related genes, gut microbiota composition, and cecal short-chain fatty acids (SCFA) in broiler chickens, which might help to identify a possible mechanism of HMB for reducing fat accumulation in broiler chickens.

## 2. Materials and Methods

### 2.1. Animals and Experimental Protocol

The experimental procedures of this study were approved by the Ethical Committee of the Institute of Subtropical Agriculture, Chinese Academy of Sciences (ISA-2020-027, 8 March 2020).

A total of 336 healthy 1-day-old male broiler chicks (Wenshi) were randomly assigned to 4 groups (six replicates per group, 14 birds per replicate). All broilers were fed one of the four diets with HMB levels of 0, 0.05, 0.10, or 0.15%. These doses of supplemental HMB were selected since it has been found in previous studies that increased average daily gain and more breast muscle yield were observed in chicks fed a 0.10% HMB diet from 21 days of age [13]. The HMB was obtained from Jiangyin TSI Pharmaceutical Co., Ltd. (Shanghai, China). The diet composition is presented in Supplementary Table S1 [14]. During the rearing period, all broilers were housed in an environmentally controlled house. The temperature was maintained at 33 °C during the first week, and then reduced by 3 °C/week until it reached 24 °C. All birds were allowed free access to diets and water.

On day 51, 12 broilers (two broilers per replicate, *n* = 6/treatment) of each treatment were randomly selected and killed after a 12 h fast to collect blood samples [14]. The abdominal fat and liver tissue samples were collected and weighed. Subsequently, the liver was snap-frozen in liquid nitrogen and stored at −80 °C for further analysis. The abdominal fat percentage was calculated as previously described [8]. The cecum contents were collected aseptically, snap frozen, and stored at −80 °C for 16S rRNA sequencing and SCFAs analysis.

### 2.2. Determination of Serum and Hepatic Lipid Profile as Well as Serum Metabolic Hormones

Blood samples were separated according to our previous studies to obtain serum [15]. Serum concentrations of triacylglycerol (TG), total cholesterol (TC), high density lipoprotein (HDL) and low density lipoprotein (LDL) were measured using a biochemical analytical instrument (Beckman CX4; Beckman Coulter, Brea, CA, USA) and commercially available kits from Roche (Basel, Switzerland). The concentrations of liver TG and TC were determined by commercial ELISA kits (Jiangsu Enzymebiao Biotechnology Co., Ltd., Jiangsu, China).

### 2.3. Measurements of Liver Fatty Acid Composition

The fatty acid composition in the liver was measured via gas–liquid chromatography of methyl esters using an Agilent 7890A GC (Agilent Technologies, Santa Clara, CA, USA) as previously described [17]. The concentrations of individual fatty acids were expressed as a percentage of total fatty acids. The following parameters were calculated based on the fatty acid composition: the sum of SFA, MUFA, PUFA, n6 PUFA, and n3 PUFA, the PUFA/SFA ratio, and the n6/n3 PUFA ratio.

### 2.4. Quantitative Real-Time PCR Analysis

The TRIzol reagent (Invitrogen, Carlsbad, California) was used to extract total RNA from liver tissue samples. The ultraviolet spectroscopy using a NanoDrop^®^ ND-1000 spectrophotometer (Thermo Fisher Scientific, Inc., Waltham, MA, USA) was used to check the RNA quantity. Thereafter, about 1.0 μg of total RNA was used to measure the mRNA expression level of lipid metabolism-related genes in the liver tissue by qRT-PCR analysis. The β-actin gene was used to normalize the expression of the target genes according to the 2^−△△Ct^ method, as previously described [18]. Primers for the target genes were designed using Primer 5.0 software and synthesized by Sangon Biotech Co., Ltd. (Shanghai, China), and their sequences are shown in Table 1.

### 2.5. Cecal Microbiome Analysis by 16S rRNA Sequencing

Microbiome DNA extracted from cecal chyme samples was used to perform cecal microbiome analysis as previously described [15]. Purified amplicons were pooled in an equimolar fashion and paired-end sequenced on an Illumina MiSeq PE300 platform/NovaSeq PE250 platform (Illumina, San Diego, CA, USA) according to the standard protocols by Majorbio Bio-Pharm Technology Co. Ltd. (Shanghai, China). Alpha diversity indices (Shannon, Simpson, Chao1, and ACE) were used to describe the diversity of the microbiome communities among samples. The order analysis for partial least squares discriminant analysis (PLS-DA) was performed to analyze the variation in the community structure between groups. Operational taxonomic units (OTUs) were further used for thr genome prediction of microbial communities by Phylogenetic Investigation of Communities by Reconstruction of Unobserved States (PICRUSt). In addition, correlation analyses between the abundances of *Bacteroidetes* and *Firmicutes*, the liver weight, and the liver concentrations of TG and TC were conducted by Pearson correlation analysis.

### 2.6. Cecal SCFAs Analysis

Cecal chyme samples (1 g) were collected to analyze the contents of cecal SCFAs, including acetic acid, propionic acid, butyric acid, isobutyric acid, valeric acid, and isovaleric acid, by using Agilent 6890 gas chromatography (Agilent Technologies, Santa Clara, CA, USA), as previously described [15].

### 2.7. Statistical Analysis

Data of this study were analyzed by one-way ANOVA using the SAS 8.2 software package, followed by a Tukey’s studentized range test to explore treatment effects. Results were presented as means ± standard errors. Orthogonal polynomial contrasts were performed to determine the linear and quadratic effects of increasing dietary HMB on the measured traits using Statistical Package for Social Sciences (SPSS) version 18.0 software. The regression analysis was performed using GraphPad Prism 7.04 software. Differences between significant means were viewed to be statistically different at *p* < 0.05. The online platform of Majorbio ISanger Cloud platform (https://cloud.majorbio.com/, 6 January 2021) was used as analysis software for microbiomes.

## 3. Results

### 3.1. Liver Weight and Abdominal Fat Percentage

**Figure 1:**
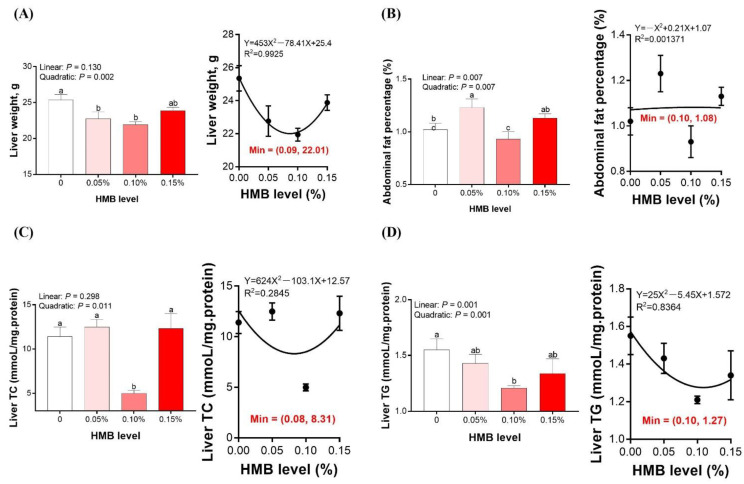
Effects of dietary HMB supplementation on liver weight (**A**), abdominal fat percentage (**B**), liver TC (**C**), and liver TG (**D**) of Wenshi broiler chickens. Data are presented as mean ± SEM (*n* = 8). ^a,b,c^ Values with different letters are significantly different among dietary HMB treatments (*p* < 0.05). HMB, beta-hydroxy-beta-methyl butyrate; TC, total cholesterol; TG, triacylglycerol.

**Figure 2:**
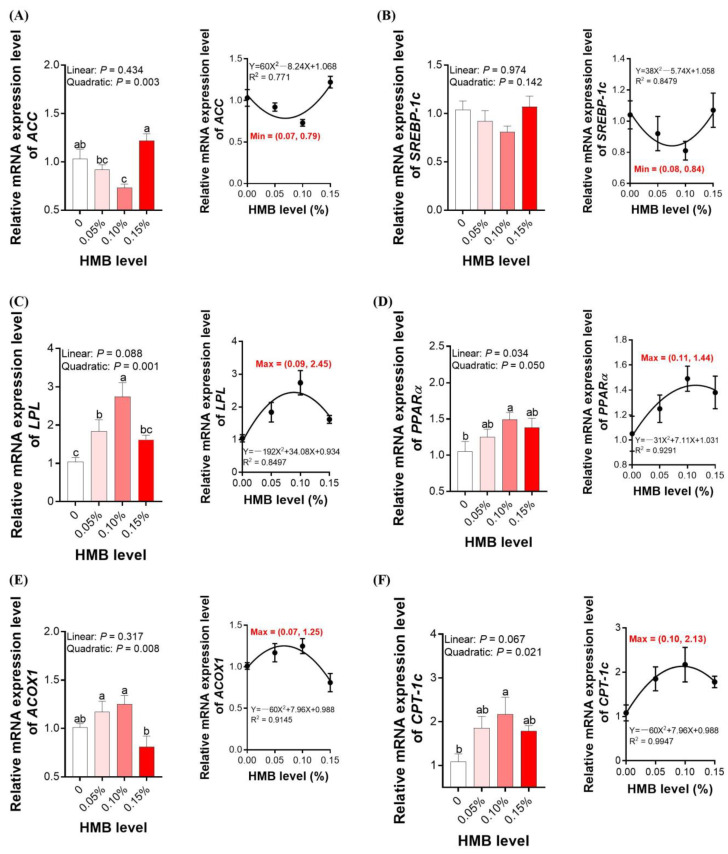
Effects of dietary HMB supplementation on the expression of lipid metabolism-related genes (**A**): ACC; (**B**), SREBP-1c; (**C**), LPL; (**D**), PPARα; (**E**), ACOX1; (**F**), CPT-1c, in the liver of broilers. Data are presented as mean ± SEM (*n* = 8). ^a,b,c^ Values with different letters are significantly different among dietary HMB treatments (*p* < 0.05). *ACC*, acetyl-CoA carboxylase; *ACOX*, acyl-CoA oxidase 1; *CPT-I* carnitine palmitoyl transferase I; HMB, beta-hydroxy-beta-methyl butyrate; *LPL*, lipoprotein lipase; *PPARα*, peroxisome proliferator activated receptor-α; *SREBP-1c*, sterol regulatory element-binding protein-1c.

In order to investigate whether HMB supplementation regulated lipid metabolism in broiler chickens, we first measured the liver weight, abdominal fat percentage, and liver TC and TG content. As shown in Figure 1A,D, both the liver weight and liver TG content were quadratically increased, with the highest values observed in the control group and the lowest values observed in the 0.10% HMB group (*p* < 0.01). The liver TC content in the 0.10% HMB group was significantly lower than in the other three groups (quadratically, *p* < 0.05, Figure 2C). Compared to the control group, the 0.05% HMB group significantly increased the abdominal fat percentage (*p* < 0.05), and the difference between the control and the other two groups was not statistically significant (*p* > 0.05, Figure 2B). According to the regression analysis, the minimum values of liver weight, abdominal fat percentage, liver TC content, and liver TG content were observed at the HMB levels of 0.09%, 0.10%, 0.08%, and 0.10%, respectively (*p* < 0.05).

### 3.2. Serum Lipid Profile

To further explore whether HMB supplementation modulated lipid metabolism in broiler chickens, serum lipid profile was analyzed. As shown in Table 2, dietary HMB supplementation quadratically decreased serum TG content, with the lowest value observed in the 0.10% HMB group (*p* < 0.05). In contrast, dietary HMB supplementation quadratically increased serum HDL-C content, with the highest value observed in the 0.10% HMB group (*p* < 0.05). No significant differences in serum concentrations of TG, LDL-C, and TC were observed among groups (*p* > 0.05).

### 3.3. Fatty Acid Composition in the Liver Tissue

Based on the abovementioned results, we found that HMB supplementation could reduce hepatic lipid accumulation in broilers. Therefore, we further analyzed whether HMB supplementation could regulate fatty acid composition in the liver. As shown in Table 3, dietary HMB supplementation greatly increased C18:3n3 PUFA concentration compared to the control group (*p* < 0.01), the maximal increase occurred at the level of 0.10%. Similarly, the C22:6n3 PUFA concentration was the highest in the 0.10% and 0.15% HMB groups and the lowest in the 0.05% HMB group, with an intermediate value observed in the control group (*p* < 0.05), but the difference between the control and 0.10% HMB group was not statistically significant. Compared to the control group, the sum of n3 PUFA was significantly increased and the ratio of n6 to n3 PUFA was significantly decreased in the 0.10% HMB group (*p* < 0.05). Dietary treatments did not significantly affect the sum of SFA, PUFA, n6 PUFA, and the PUFA:SFA ratio (*p* > 0.05).

### 3.4. Lipid Metabolism-Related Genes Expression in the Liver

To investigate whether HMB supplementation regulated hepatic lipid accumulation through modulating the gene expression implicated in fatty acid metabolism, the mRNA expression of genes implicated in fatty acid synthesis (*ACC* and *SREBP-1c*) and fat deposition (*ACOX*, *CPT-I*, *LPL*, and *PPARα*) was determined. As shown in Figure 2A, the mRNA level of *ACC* in the livers of broilers treated with 0.10% HMB was significantly decreased relative to the control (*p* < 0.05). According to the regression analysis, the minimum expression of *ACC* occurred at the HMB level of 0.07% (*p* < 0.05). In contrast, the expression levels of hepatic *CPT-I*, *LPL*, *ACOX1*, and *PPARα* were significantly increased in the 0.10% HMB-treated broilers compared to those of the control group (*p* < 0.05, Figure 2B–F). According to the regression analysis, the maximum expression of *LPL*, *PPARα*, *ACOX1*, and *CPT-I* occurred at the HMB level of 0.09%, 0.11%, 0.07%, and 0.10%, respectively (*p* < 0.05). However, HMB treatments did not alter *SREBP-1c* mRNA expression.

### 3.5. Cecal Microbiota

**Figure 3:**
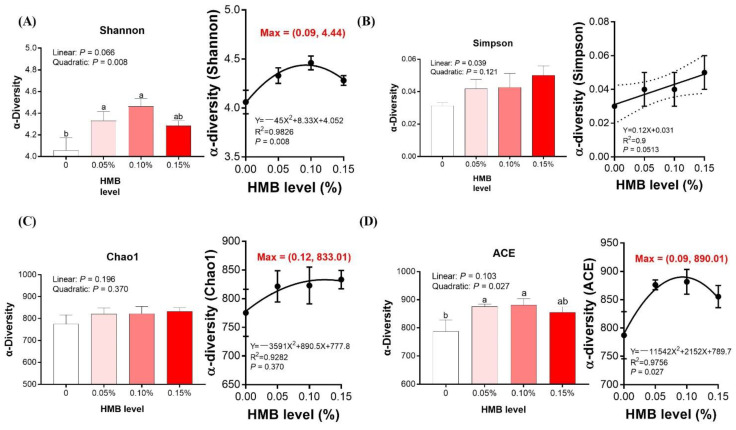
Alpha diversity metrics of cecal bacterial communities. (**A**) Histogram for comparison of species diversity (Shannon index), (**B**) Histogram for comparison of species diversity (Simpson index), (**C**) Histogram for comparison of species richness (Chao1 index), (**D**) Histogram for comparison of species richness (ACE index). Data are presented as mean ± SEM (*n* = 8). ^a,b,c^ Values with different letters are significantly different among dietary HMB treatments (*p* < 0.05).

**Figure 4:**
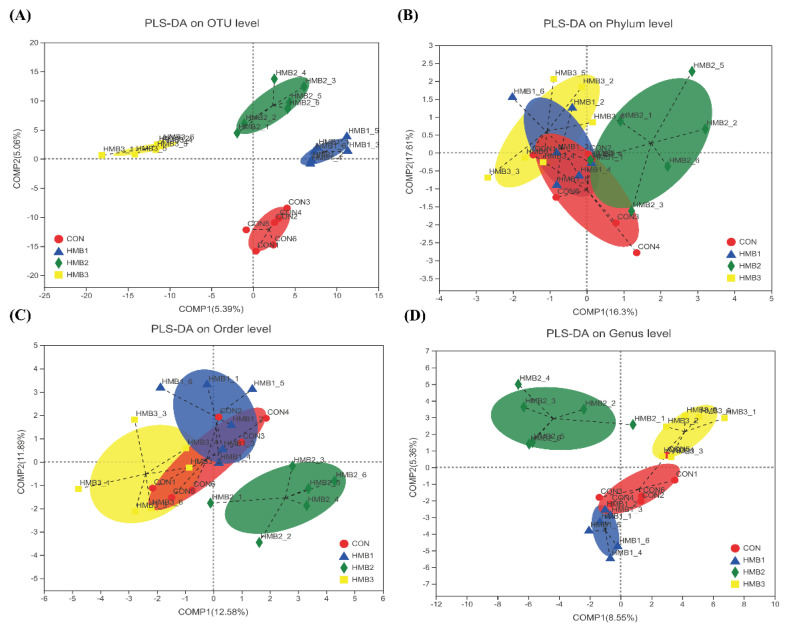
PLS-DA on OUT level (**A**), phylum level (**B**), order level (**C**), and genus level (**D**). HMB1 = 0.05% HMB; HMB2 = 0.10% HMB; HMB3 = 0.15% HMB.

**Figure 5:**
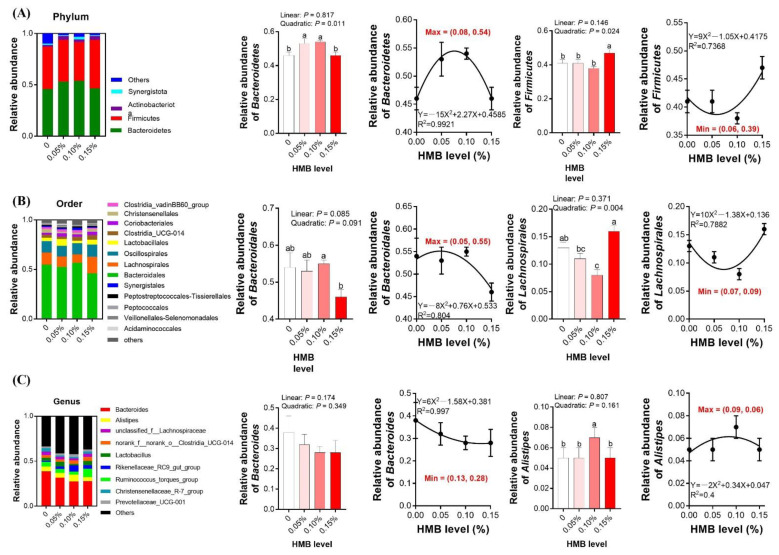
Effects of dietary HMB supplementation on the cecal microbiota. Microbiota compositions at the phylum level (**A**), microbiota compositions at the order level (**B**), microbiota compositions at the genus level (**C**). Data are presented as mean ± SEM (*n* = 8). ^a,b,c^ Values with different letters are significantly different among dietary HMB treatments (*p* < 0.05).

Gut microbiota are closely related to lipid metabolism, hence we investigated the composition of the cecal microbiota in broiler chickens. After size filtering, quality control, and chimera removal, 68,137 clean tags were subjected to the following analysis and clustered into OTUs (Supplementary Table S2). As presented in Figure 3A,D, the indexes of both Shannon and ACE were quadratically increased, with the highest value observed in the 0.10% HMB group (*p* < 0.05), but the value was not significantly greater than the other treatment groups. According to the regression analysis, the maximum values of both the Shannon and ACE indexes were obtained at the HMB level of 0.09% (*p* < 0.05). No observable difference in the indexes of Simpson and Chao1 were noted upon dietary treatment (*p* > 0.05, Figure 3B,C). As shown in Figure 4, samples collected from HMB chickens were significantly separated from those of control chickens in the PLS-DA analysis on the OUT and genus levels. However, on the phylum and order levels, they were barely separated. As illustrated in Figure 5A, chickens among the groups exhibited a distinct clustering of microbial communities at the phylum level. The *Bacteroidetes* abundance was quadratically increased, with the highest value observed in the 0.10% HMB group (*p* < 0.05). The *Firmicutes* richness in the 0.15% HMB group was higher than that in the other three groups (quadratically, *p* < 0.05). According to the regression analysis, the maximum abundance of *Bacteroidetes* and the minimum abundance of *Firmicutes* were obtained at the HMB levels of 0.08% and 0.06%, respectively (*p* < 0.05). The order level analysis indicated that HMB tended to increase the abundance of *Bacteroidetes* (linearly and quadratically, 0.05 < *p* < 0.10, Figure 5B), with the highest value observed in the 0.10% HMB group. By contrast, HMB significantly decreased the richness of *Lachnospirales* (quadratically, *p* < 0.05, Figure 5B), with the lowest value observed in the 0.10% HMB group. At the genus level, the beneficial bacteria *Alistipes* was significantly enriched in the 0.10% HMB group (*p* < 0.05, Figure 5C). According to the regression analysis, the maximum abundance of *Alistipes* was obtained at the HMB level of 0.09% (*p* < 0.05).

### 3.6. Cecal SCFAs Concentrations

**Figure 6:**
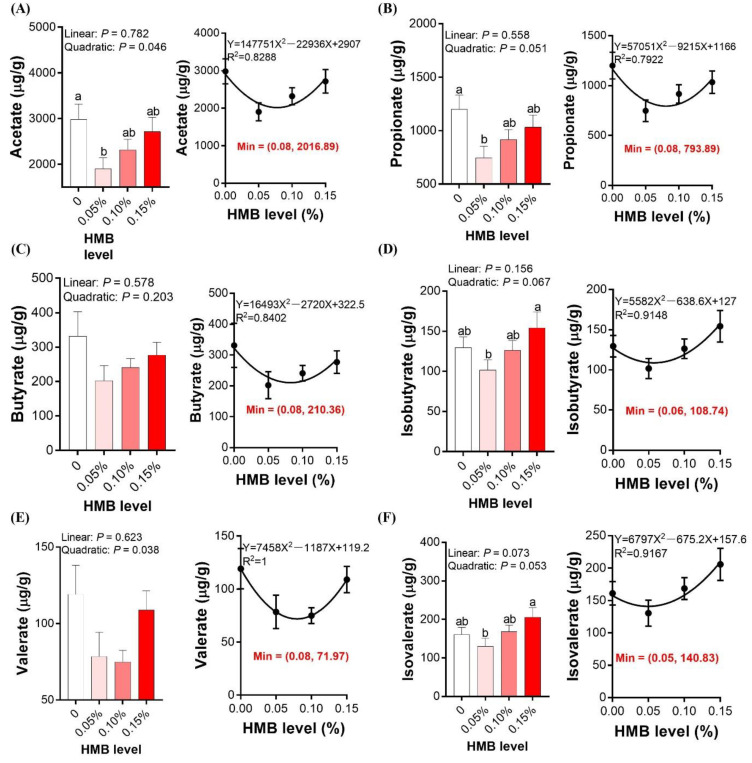
Effects of dietary HMB supplementation on the cecal concentrations of short-chain fatty acids ((**A**), acetate; (**B**), propionate; (**C**), butyrate; (**D**), isobutyrate; (**E**), valerate; (**F**), isovalerate). Data are presented as mean ± SEM (*n* = 8). ^a,b,c^ Values with different letters are significantly different among dietary HMB treatments (*p* < 0.05).

To further examine whether HMB affected the microbiota metabolites, we measured cecal SCFAs in broiler chickens. As shown in Figure 6, the acetate concentration was the highest in the control group and lowest in the 0.05% HMB group, with intermediate values in the other two groups (quadratically, *p* < 0.05). Similar alterations were observed for the propionate concentration (quadratically, *p* = 0.051). The concentrations of isobutyrate and isovalerate were the highest in the 0.15% HMB group and lowest in the 0.05% HMB group, with intermediate values observed in the other two groups (quadratically, 0.05 < *p* < 0.10). No significant differences in the concentrations of butyrate and valerate were observed among the groups (*p* > 0.05).

### 3.7. Correlation Analysis

**Figure 7:**
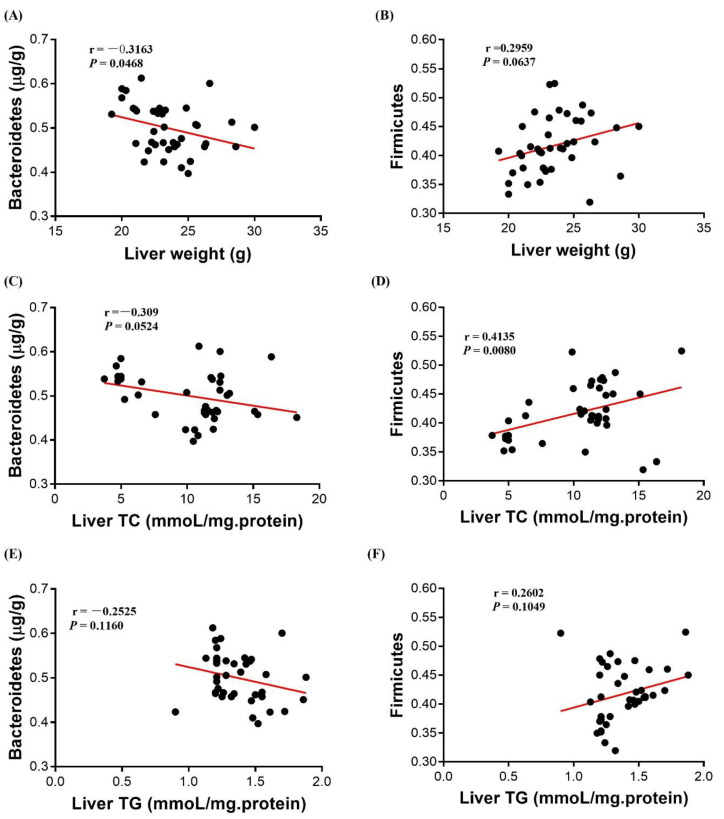
Correlation analyses between lipid metabolism related-parameters (liver weight, liver TG and TC) and the relative abundances of *Bacteriodetes* and *Firmicutes*, respectively (*n* = 32). (**A**) the correlation between liver weight and the relative abundance of *Bacteriodetes*; (**B**) the correlation between liver weight and the relative abundance of *Firmicutes*; (**C**) the correlation between liver TC and the relative abundance of *Bacteriodetes*; (**D**) the correlation between liver TC and the relative abundance of *Firmicutes*; (**E**) the correlation between liver TG and the relative abundance of *Bacteriodetes*; (**F**) the correlation between liver TG and the relative abundance of *Firmicutes*. TC, total cholesterol; TG, triacylglycerol.

In order to investigate whether the regulatory effects of HMB on hepatic lipid accumulation was due to the alteration of gut microbiota, we performed correlation analyses between the relative abundances of *Bacteroidetes* and *Firmicutes* and lipid accumulation- related parameters (liver weight, liver TC and TG content). As presented in Figure 7, the *Bacteroidetes* abundance was negatively correlated with the liver weight (*p* < 0.05) and tended to be negatively correlated with the liver TC concentration (*p* = 0.0524). By contrast, the *Firmicutes* abundance tended to be positively correlated with the liver weight (*p* = 0.0637) and was positively correlated with the liver TC concentration (*p* < 0.05). However, there was no significant correlation between the liver TG concentration and the abundance of *Bacteroidetes* or *Firmicutes* (*p* > 0.05).

### 3.8. PICRUSt Functional Prediction Analysis

**Figure 8:**
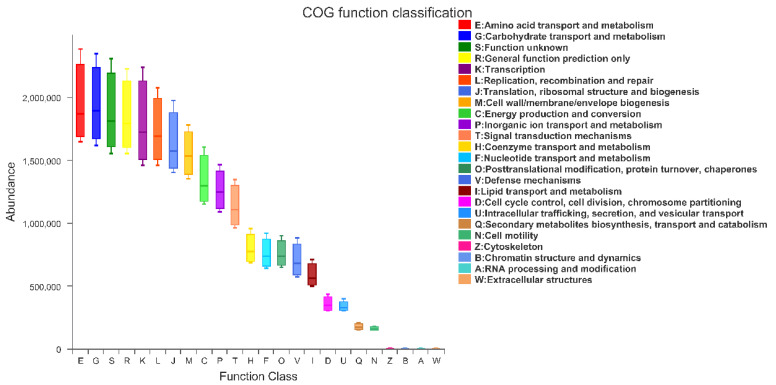
Variance of COG based function abundances in all samples.

To investigate the effect of HMB supplementation on cecal bacterial function, we used PICRUSt (phylogenetic investigation of communities by reconstruction of unobserved states) to perform bacterial function prediction analysis. Based on the Cluster of Orthologous Groups (COG) database, we obtained a microbial COG profile and correlated the microbial functional features with the key enzymes found in the broiler samples. As shown in Figure 8, the metabolic functions were enriched in our samples, suggesting that the microbial metabolism in the broiler samples tended to be vigorous. In particular, these functional features included: amino acid transport and metabolism; carbohydrate transport and metabolism; general function prediction only; transcription; replication, recombination and repair; translation, ribosomal structure and biogenesis; cell wall/membrane/envelope biogenesis; energy production and conversion; inorganic ion transport and metabolism; and signal transduction mechanisms.

## 4. Discussion

HMB is a metabolite of leucine and is widely used as a nutritional agent in the field of poultry production to improve growth and to reduce mortality [13,19,20]. Here, we used broiler chickens as model of choice to elucidate the effects of HMB on hepatic fat metabolism. We found that 0.10% HMB supplementation could inhibit fat deposition in the liver of broiler chickens, as manifested by the significantly reduced liver weight and the significantly reduced liver TG and TC concentrations. Our current results fit well with previous studies reporting that HMB alleviated fat accumulation in the liver of high fat diets-fed mice [15]. It is well known that excessive fat deposition in the liver might give rise to metabolic diseases and result in adverse health effects for consumers. Therefore, it appears that broiler chickens might benefit from HMB due to the inhibition of hepatic fat accumulation [21].

Serum TG, TC, HDL-C, and LDL-C are the key biochemical parameters reflecting the status or the rate of lipid metabolism. Lipid accumulation in the form of TG is considered to be a sensitive index or measure of a tissue’s exposure to fatty acids [22]. Current studies have shown that 0.10% HMB supplementation significantly decreased serum TG content and increased serum HDL-C content. The reduced serum TG levels in the 0.10% HMB group indicated that the lipid content in the liver tissue was decreased, as evidenced by significantly decreased liver TG and TC contents. HDL-C is mainly responsible for transporting the cholesterol from peripheral tissue to liver, where cholesterol is converted to bile acids, bile salts, and cholesteryl esters [23]. Therefore, higher levels of HDL-C in the serum of 0.10% HMB-treated broilers might indicate a relatively balanced lipid metabolism. In addition, evidence indicates that C22:6 n3 could increase serum HDL-C content and decrease TG content [24], thus inhibiting lipid accumulation [25]. Therefore, it raises the possibility that the improved lipid metabolism in 0.10% HMB-treated broilers might be related to an increase in hepatic n3 PUFAs, especially C22:6n3 [26]. Therefore, this study provides evidence that feeding HMB to broilers leads to substantial decreases in liver fat accumulation and promotes n3 PUFAs incorporation into hepatic lipid pools, thus enhancing the nutritional benefits of animal products.

In avian species, the liver is the most important organ for the intermediary metabolism of lipids and energy, since lipogenesis mainly occurrs in this organ [27]. Moreover, hepatic lipogenesis can be highly responsive to nutritional intervention, and hence the regulation of hepatic gene expression may exert a key role in modulating fat metabolism of broilers [28,29]. To elucidate the mechanism of the lipid lowering effects of HMB, we analyzed the expression levels of genes related to synthesis and metabolism of fatty acids in the liver of broiler chickens. ACC is a rate-limiting enzyme of fatty acid de novo synthesis [30,31]. PPARα, ACOX, and CPT-1 are key mediators in the control of mitochondrial and peroxisomal fatty acid oxidation, as well as fatty acid β-oxidation [32,33]. Our data showed that decreased expressions of ACC as well as increased expressions of PPARα, ACOX, and CPT-1 were found in broilers treated with 0.10% HMB. Overall, the current studies demonstrated that HMB treatment could regulate lipid metabolism in the liver of broilers partially by inhibiting fatty acid *de novo* synthesis and promoting fatty acid β-oxidation.

In recent years, it has been demonstrated that dietary treatments induced-alteration of gut microbiota exerts important roles in modulating lipid metabolism [8,34,35]. Our data showed that Shannon index to assess diversity was significantly different between 0.10% HMB-treated group and control group, suggesting a deep alteration in microbial diversity. To examine whether dietary HMB supplementation inhibited lipid metabolism in broilers via gut microbiota, we performed high-throughput sequencing on hypervariable region of the 16S rRNA genes of cecal bacteria. At the phylum level, dietary supplementation with HMB increased the *Bacteriodetes* relative abundance. *Bacteriodetes* exerts a key role in carbohydrate fermentation and can ferment carbohydrate to produce a variety of volatile fatty acids, which are utilized as an energy source by the host [36]. Our data showed that the reduction in fat deposition, as manifested by reduced liver TG and TC as well as liver weight, was related to increased *Bacteriodetes* and decreased *Firmicutes* populations. These results are well-matched previous observations made in other work, showing that gut microbiota exerts key roles in liver lipid metabolism [37,38,39,40]. At the genus level, the HMB group shower higher abundance of *Alistipes* than the control group. *Alistipes* has been regarded as beneficial bacteria, which have lipid metabolism-regulation capabilities [8,41]. Therefore, it is posited that HMB supplementation leads to the growth of some beneficial bacteria and the inhibition of some harmful bacteria. Therefore, our results demonstrate that dietary HMB supplementation-induced reduction of chicken fat deposition is at least partially associated with improvements in the relative population densities of the gut microbiome.

In animal production industries, it is of great importance to convert food into body weight, and the gut microbiota exert a critical role in the efficiency of energy extraction from diets and host metabolism [42,43]. Moreover, accumulating and emerging lines of evidence have revealed a positive association between the microbiota diversity in cecum, where the chyme is largely fermented, and growth performance in broilers [43,44]. Similarly in our study, we found that higher richness of microbiota in cecum is accompanied by higher growth performance in broilers [14]. Furthermore, we performed PICRUSt analysis to reveal alterations in metabolism triggered by these bacteria, and demonstrated that carbohydrate transport and metabolism was the primary factor that promoted growth. Therefore, we speculated that improvements in gut microbiota in response to HMB supplementation promoted more efficient absorption of calories and subsequent weight gain.

## 5. Conclusions

In conclusion, dietary HMB supplementation increased n3 PUFA and decreased fat deposition in the livers of broiler chickens, and these effects might be associated with alterations in the composition of gut microbiome. These findings suggest that HMB could be used as a feed additive to regulate gut microbiota and suppress fat deposition in chickens.

## Figures and Tables

**Table 1:** Primers used for real-time PCR.

Genes ^1^	Primers	Sequences (5′-3′)	Size (bp)
*ACC*	Forward	GCTGGGTTGAGCGACTAATG	173
	Reverse	GGGAAACTGGCAAAGGACTG	
*LPL*	Forward	TCATTGTTGTGGACTGGC	139
	Reverse	TGGACATTGTTGAGAGGGT	
*SREBP-1c*	Forward	CCTGGAAGAAGCATCATGGC	181
	Reverse	AGAGCACAGAGGATTCGGAG	
*PPARα*	Forward	TGTGGAGATCGTCCTGGTCT	103
	Reverse	CGTCAGGATGGTTGGTTTGC	
*ACOX1*	Forward	ATGTCACGTTCACCCCATCC	133
	Reverse	AGGTAGGAGACCATGCCAGT	
*CPT-1*	Forward	GAAGACGGACACTGCAAAGG	223
	Reverse	GGGCAAGTTGAATGAAGGCA	
*β-actin*	Forward	GTGTGATGGTTGGTATGGGC	225
	Reverse	CTCTGTTGGCTTTGGGGTTC	

^1^ The abbreviations of the gene names are shown as follows: *ACC*: acetyl-CoA carboxylase; *ACOX1*, acyl-CoA oxidase 1; *CPT-1*: carnitine palmitoyltransferase-1; *LPL*: lipoprotein lipase; *PPARα*: peroxisome proliferator activated receptor alpha; *SREBP-1c*: sterol regulatory element-binding protein-1c.

**Table 2:** Effects of HMB on serum lipid profile and metabolic hormones in broiler chickens.

Items ^1^	Dietary Levels of HMB, %				SEM	*p*-Value ^1^		
	0	0.05	0.10	0.15		ANOVA	Linear	Quadratic
TG, mmol/L	0.45 ^a^	0.45 ^ab^	0.38 ^c^	0.41 ^bc^	0.07	0.06	0.006	0.018
TC, mmol/L	3.02	3.20	3.00	2.96	0.19	0.413	0.422	0.428
LDL-C, mmol/L	0.72	0.66	0.65	0.70	0.16	0.884	0.806	0.718
HDL-C, mmol/L	2.07 ^b^	2.24 ^ab^	2.33 ^a^	2.14 ^ab^	0.15	0.050	0.367	0.024

^1^ TG, triglycerides; TC, total cholesterol; LDL-C, low-density lipoprotein- cholesterol; HDL-C, high-density lipoprotein- cholesterol. ^a,b,c^ Values (*n* = 8) within a row with different superscripts differ significantly (*p* < 0.05).

**Table 3:** Hepatic fatty acid composition in broilers fed diets with various levels of HMB (% of total fatty acids).

Items	Dietary Levels of HMB, %				SEM	*p*-Value ^1^		
	0	0.05	0.10	0.15		ANOVA	Linear	Quadratic
C10:0	0.24	0.24	0.23	0.22	0.08	0.84	0.37	0.66
C12:0	0.16 ^a^	0.15 ^ab^	0.14 ^b^	0.13 ^b^	0.05	0.04	<0.01	0.02
C14:0	0.14 ^b^	0.12 ^b^	0.19 ^a^	0.16 ^ab^	0.07	0.02	0.12	0.24
C16:0	16.70	16.17	17.09	16.04	0.42	0.44	0.65	0.80
C16:1	0.35 ^c^	0.61 ^b^	1.01 ^a^	0.74 ^b^	0.17	<0.01	<0.01	<0.01
C17:0	0.17 ^ab^	0.16 ^b^	0.19 ^a^	0.17 ^ab^	0.05	0.06	0.24	0.49
C18:0	24.67	24.69	22.88	22.85	0.47	0.06	0.02	0.06
C18:1 n9t	0.10	0.10	0.10	0.08	0.05	0.15	0.03	0.08
C18:1 n9c	5.32 ^b^	6.00 ^b^	6.20 ^b^	7.54 ^a^	0.38	0.01	<0.01	<0.01
C18:2 n6c	21.42 ^ab^	22.44 ^a^	20.88 ^b^	20.81 ^b^	0.39	0.05	0.11	0.15
C18:3 n6	0.13 ^c^	0.17 ^a^	0.17 ^ab^	0.15 ^b^	0.05	<0.01	0.16	<0.01
C20:1	0.20	0.17	0.18	0.18	0.06	0.18	0.23	0.29
C18:3 n3	0.27 ^b^	0.47 ^a^	0.58 ^a^	0.43 ^a^	0.14	<0.01	0.05	<0.01
C20:2	0.70 ^ab^	0.62 ^b^	0.75 ^a^	0.74 ^a^	0.10	0.02	0.10	0.14
C22:0	0.24	0.25	0.25	0.27	0.07	0.69	0.26	0.48
C20:3 n6	1.21	1.39	1.36	1.33	0.18	0.55	0.43	0.38
C20:4 n6	19.29	19.63	18.17	20.30	0.71	0.76	0.81	0.79
C24:0	0.64 ^b^	0.93 ^a^	0.80 ^ab^	0.75 ^ab^	0.16	0.06	0.56	0.08
C22:6 n3	4.34 ^ab^	4.00 ^b^	4.75 ^a^	4.64 ^a^	0.25	0.02	0.06	0.14
SFA ^2^	42.95	42.70	41.76	40.59	0.52	0.15	0.02	0.07
MUFA^3^	5.98 ^c^	6.88 ^bc^	7.50 ^ab^	8.54 ^a^	0.40	<0.01	<0.01	<0.01
PUFA ^4^	47.35	48.71	48.57	48.39	0.71	0.90	0.87	0.98
∑PUFA:SFA	1.11	1.14	1.17	1.19	0.12	0.60	0.22	0.41
∑n6 PUFA ^5^	42.04	43.63	40.57	42.59	0.68	0.44	0.81	0.96
∑n3 PUFA ^6^	4.61 ^bc^	4.47 ^c^	5.32 ^a^	5.07 ^ab^	0.25	<0.01	0.01	0.04
∑n6:n3 PUFA	9.12 ^ab^	9.80 ^a^	7.67 ^c^	8.53 ^bc^	0.33	<0.01	0.03	0.10

^1^ Duncan, linear, and quadratic effects for HMB inclusion levels, ^2^ SFA = C10:0 + C12:0 + C14:0 + C16:0 + C17:0 + C18:0 + C22:0 + C24:0, ^3^ MUFA = C16:1 + C18:1n9t + C18:1n9c + C20:1, ^4^ PUFA = C18:2n6c + C18:3n6 + C18:3n3 + C20:2 + C20:3n6 + C20:4n6 + C22:6n3, ^5^ n3 PUFA = C18:3n3 + C22:6n3, ^6^ n6 PUFA = C18:2n6c + C18:3n6 + C20:3n6 + C20:4n6, ^a,b,c^ Values (*n* = 8) within a row with different superscripts differ significantly (*p* < 0.05).

## Data Availability

The data presented in this study are available on request from the corresponding author.

## Supplementary Materials

The following supporting information can be downloaded at: https://www.mdpi.com/article/10.3390/microorganisms10010169/s1, Table S1: Composition and nutrient content of basal diets of broilers (air-dry basis, %); Table S2: Effects of HMB on valid reads of microbiota.
