# Supplementary material for: Dietary Beta-Hydroxy-Beta-Methyl Butyrate Supplementation Inhibits Hepatic Fat Deposition via Regulating Gut Microbiota in Broiler Chickens
Source: Microorganisms. 2022 Jan 13;10(1):169. doi: 10.3390/microorganisms10010169 (PMC8781658; doi:10.3390/microorganisms10010169)
Supplement: Supplementary file 1 [file microorganisms-10-00169-s001.zip › microorganisms-1484003-SI.pdf]

Supplementary Table S1. Composition and nutrient content of basal diets of broilers (air-dry basis, %)

**Item**	Starter phase	Grower phase
	d 1 to 21	d 22 to 51
Ingredients, %		
Corn	56.49	63.45
Soybean meal	29.63	26.63
Corn gluten meal	0.70	0.32
Wheat bran	4.60	1.00
Soybean oil	4.40	4.00
L-Lysine HCl	0.26	0.26
DL-methionine	0.29	0.38
Threonine	0.09	0.10
Calcium hydrogen phosphate	1.70	2.00
Limestone	1.36	1.36
Premix[1]	0.48	0.50
Nutrients (calculated value)		
ME, MJ/kg	12.54	12.78
Crude protein	19.38	17.94
Calcium	1.00	1.05
Total phosphorus	0.66	0.68
Lysine	1.16	1.08
Methionine	0.59	0.66
Methionine+cystine	0.92	0.96

[1] The premix provided per kilogram of diet: Vitamin A 12,500 IU; Vitamin D_3 2,500 IU; Vitamin E 18.75 mg; Vitamin K_3 2.65 mg; Vitamin B_1 2 mg; Vitamin B_2 6 mg; Vitamin B_{12} 25 µg; pantothenic acid 12 mg; nicotinic acid 50 mg; biotin 32.5 µg; folic acid 1.25 mg; iron 80 mg; zinc 75 mg; manganese 100 mg; iodine 0.35 mg; copper 8 mg; selenium 0.15 mg.

Supplementary Table S2. Effects of HMB on valid reads of microbiota.

#Sample_name	Valid_reads(#)	AvgLen(nt)	Base(nt)	Q30	Q20
Con1	70489	420	29606576	94.13	98.01
Con2	70011	413	28921786	93.55	97.76
Con3	71824	413	29679463	93.43	97.70
Con4	53713	414	22242389	93.55	97.78
Con5	72155	420	30299565	93.53	97.60
Con6	74971	420	31454006	93.14	97.58
Con7	68861	417	28700631	93.55	97.74
Con8	67461	417	28700631	93.55	97.74
HMB0.5%-1	70726	420	29674818	93.98	97.92
HMB0.5%-2	71196	419	29813809	93.37	97.53
HMB0.5%-3	67251	418	28105361	93.72	97.85
HMB0.5%-4	72625	415	30108802	93.65	97.80
HMB0.5%-5	50638	416	21061527	93.69	97.86
HMB0.5%-6	74950	419	31393628	93.38	97.66
HMB0.5%-7	67898	418	28359658	93.63	97.77
HMB0.5%-8	68938	418	28359658	93.63	97.77
HMB1.0%-1	70774	417	29499725	93.52	97.56
HMB1.0%-2	70217	418	29364633	93.36	97.66
HMB1.0%-3	69565	418	29076645	93.95	97.89
HMB1.0%-4	62434	416	25958340	93.12	97.60
HMB1.0%-5	57158	415	23728143	93.51	97.75
HMB1.0%-6	67953	419	28478148	93.04	97.55
HMB1.0%-7	66350	417	27684272	93.42	97.67
HMB1.0%-8	65010	417	27684272	93.42	97.67
HMB1.5%-1	69271	412	28523691	93.94	97.96
HMB1.5%-2	73600	420	30886020	94.35	98.09
HMB1.5%-3	73707	418	30784718	93.42	97.73
HMB1.5%-4	69653	418	29098458	93.15	97.44
HMB1.5%-5	70540	414	29178205	93.30	97.66
HMB1.5%-6	59876	414	24774283	93.89	97.93
HMB1.5%-7	69441	416	28874229	93.67	97.80
HMB1.5%-8	71145	416	28874229	93.67	97.80
